# Structure Sensitive Reaction Kinetics of Chiral Molecules on Intrinsically Chiral Surfaces

**DOI:** 10.1021/acs.jpcc.4c04224

**Published:** 2024-08-13

**Authors:** Kareem Abdelmaqsoud, Michael Radetic, Carlos Fernández-Cabán, Michael Widom, John R. Kitchin, Andrew J. Gellman

**Affiliations:** ^†^Department of Chemical Engineering and ^‡^Department of Physics, Carnegie Mellon University, 5000 Forbes Ave, Pittsburgh, Pennsylvania 15213, United States

## Abstract

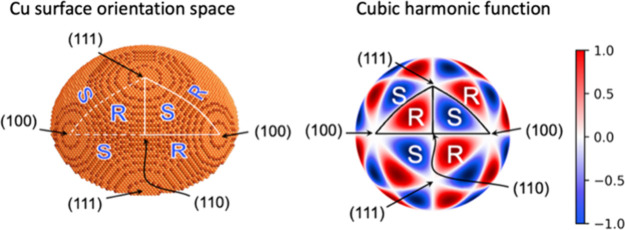

Enantiospecific heterogeneous catalysis utilizes chiral surfaces to resolve enantiomers via structure sensitive surface chemistry. The catalyst design challenge is the identification of chiral surface structures that maximize enantiospecificity. Herein, we develop data driven models for the enantiospecificity of tartaric acid reactions on chiral Cu(*hkl*)^R&S^ surfaces. Measurements of enantiospecific rate constants were obtained by using curved Cu(*hkl*)^R&S^ surfaces that enable kinetic measurements on hundreds of chiral surface orientations. One model uses feature vectors derived from generalized coordination numbers to capture the local structure around Cu atoms exposed by the Cu(*hkl*)^R&S^ surfaces. The second model introduces the use of chiral cubic harmonic functions to capture the symmetry constraints of the face-centered cubic Cu structure. The model using 58 generalized coordination numbers has a fitting error similar to that of the model using only 5 cubic harmonic functions. The two models predict maxima in the enantiospecificity on surfaces with very similar surface orientations. The models developed in this work are applicable for any enantiospecific reaction happening on any chiral material with a cubic lattice structure, opening the way to understanding the surface structure sensitivity of the enantiospecific reaction kinetics.

## 1 Introduction

The kinetics of chemical reactions on catalytic surfaces are significantly influenced by the surface atomic structure.^1^ One of the goals of surface chemistry and catalysis is to understand the relationship between the surface structure and surface reaction kinetics. Catalytic ammonia synthesis on Fe single-crystal surfaces is an example of a structure sensitive catalytic processes for which different surface structures lead to vastly different kinetics.^2^ Some of the most subtle manifestations of surface structure sensitivity are observed in the domain of enantiospecific heterogeneous catalysis. A chiral molecule has nonsuperimposable mirror images called enantiomers. Enantiospecificity arises when a chiral catalyst surface imparts different reaction kinetics to adsorbed enantiomers, thereby enabling the reactive resolution of one enantiomer from the other.^3^ In this work, we develop models for the structure sensitive reaction kinetics of chiral molecules on intrinsically chiral surfaces.

In addition to being a fundamental scientific goal of catalytic surface chemistry, understanding the enantiospecific kinetics of reactions on chiral surfaces has great practical importance, particularly in the pharmaceutical industry.^4^ The two enantiomers of a chiral drug molecule can induce vastly different physiological effects. For instance, Thalidomide is a chiral drug that was administered to pregnant women in the late 1950s as a racemic (equimolar) mixture of its two enantiomers R(S).^5,6^ The R-enantiomer of Thalidomide reduced morning sickness, but the S-enantiomer was found to cause birth defects. This highlighted the critical need for enantiopure chiral pharmaceuticals produced using enantioselective synthesis and purification processes. These processes require chiral catalytic surfaces to differentiate between the two enantiomers of the chiral pharmaceuticals. The challenge in developing these processes is designing chiral surfaces with structures that optimize the enantioselectivity to the desirable product.

Many chiral catalytic surfaces have been prepared by modifying metal surfaces with chiral adsorbates.^7,8^ In spite of the fact that bulk metals have achiral structures, they can in fact expose chiral surface planes.^9,10^ Metal surfaces expose atomic structures derived from the bulk structure of the metal crystal and the orientation of the surface normal relative to the metal unit cell vectors. The (100) surface of a face-centered-cubic (FCC) metal has atoms arranged in a square array, while the (111) surface has atoms arranged in a hexagonal array. Because these two surfaces exhibit structures with mirror symmetry, they are achiral. Metal surfaces with structures based on flat terraces separated by kinked step edges do not exhibit mirror symmetry. Thus, they are intrinsically chiral surfaces. The first experimental evidence of intrinsic chirality was observed for the enantiospecific electro-oxidation of d- and l-glucose on chiral Pt(643) surfaces in 1999.^11^ Since then, many intrinsically chiral metal surfaces have been shown to exhibit enantiospecific interactions with chiral adsorbates.^9,12−15^ Chiral surface structures having highly enantiospecific interactions with chiral adsorbates will, in principle, lead to highly enantiospecific reactions and processes. Therefore, understanding the chiral adsorbate reaction kinetics on chiral surfaces is essential for designing effective catalysts for enantiospecific surface chemistry. Herein, we build models to predict the enantiospecificity as a function of the structure of metallic surfaces. These models will help in understanding what makes one surface structure more enantiospecific than another.

**Figure 1:**
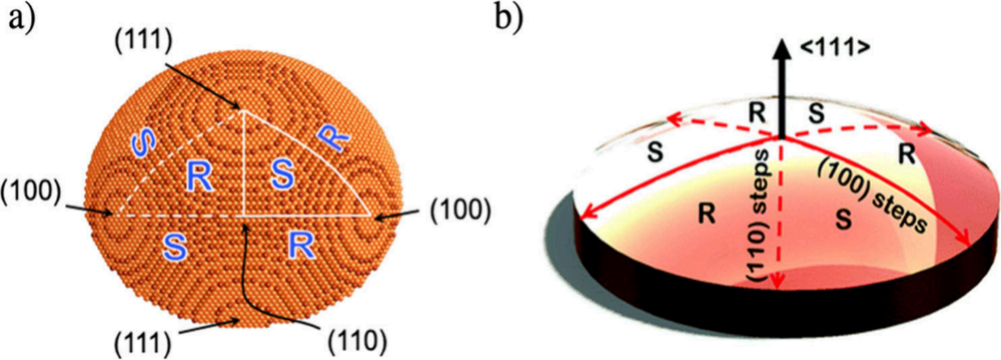
(a) Illustration of a spherical nanocrystal with FCC bulk structure. The two triangles highlighted by the white edges are examples of stereographic triangles. They contain chiral surfaces with opposite handedness R(S). Reproduced from Gellman.^9^ Copyright 2021 American Chemical Society. (b) Illustration of a Cu(111) ± 14°-S^4^C. The (111) plane is exposed at the center point. Along the solid red lines, the S^4^C surface exposes achiral surfaces with (111) terraces and (100) steps. Along the dashed red lines, the S^4^C surface exposes achiral surfaces with (111) terraces and (110) steps. The Cu(111) surface has six regions of alternating R- or S-chirality. Reproduced from Fernández-Cabán et al.^21^ Available under a CC-BY 3.0 license. Copyright 2022 Royal Society of Chemistry.

For an achiral bulk metal, one can specify an infinite number of surface orientations that are intrinsically chiral. A spherical FCC crystal such as the one shown in Figure 1a exposes all of the possible surface structures. Because of the high symmetry of the FCC crystal, the surface of the sphere is covered by 48 triangular regions that expose a continuous range of surface orientations lying between the low Miller index directions. These are called stereographic triangles. The surface orientations at the vertices of these triangles are the high-symmetry low Miller index planes, (100), (110), and (111). These surface orientations are achiral. The points along the edges of the triangles expose surfaces with structures based on low-Miller index terraces separated by monatomic step edges. These are all high symmetry surface orientations and are therefore achiral. The points inside the triangles expose surfaces with structures based on low Miller index terraces separated by kinked step edges. These are low symmetry surfaces that lack a mirror plane, and therefore, they are chiral and exist as R(S) enantiomers. The surface of the sphere is covered by 24 triangles with R handedness and 24 triangles with S handedness. Since all stereographic triangles expose identical surfaces, it is sufficient to model the enantiospecificity of surfaces that belong to one of the R or S triangles. The challenge in building such a model is collecting experimental kinetic data across many surface structures that span the stereographic triangle.

Surface structure spread single crystals (S^4^Cs) are curved crystal surfaces that expose a continuous distribution of surface orientations, enabling the study of structure dependent surface properties. Figure 1b shows a rendering of a S^4^C sample that is used to collect kinetic data on a continuous range of surface orientations that lie vicinal to the (111) direction. The six high symmetry directions divide the S^4^C into 3 regions with R handedness and 3 regions with S handedness. Combined with spatially resolved experimental surface analysis techniques, S^4^Cs allow the study of the enantiospecific surface reaction kinetics of chiral adsorbates. By employing multiple S^4^Cs that span the stereographic triangle, it becomes possible to study the enantiospecificity across the entire surface orientation space.

This work focuses on the enantiospecific decomposition kinetics of tartaric acid (TA, HO_2_CCH(OH)CH(OH) CO_2_H) enantiomers on continuous spreads of chiral copper surface orientations. TA is an ideal chiral probe for studying enantiospecific surface chemistry because of its well-understood decomposition mechanism on Cu surfaces.^16−20^ TA decomposes upon heating into CO_2_, CO, H_2_O, H_2_ and leaves some C and O atoms adsorbed to the surface.^21^ TA undergoes decomposition via a vacancy-mediated explosive reaction mechanism characterized by a slow initiation step followed by a rapid explosion step.^22^Equation 1 is the rate law for this reaction where θ_*TA*_^(*hkl*)^ is the TA coverage of the surface, *k*_*i*_^(*hkl*)^ is the initiation rate constant, and *k*_*e*_^(*hkl*)^ is the explosion rate constant on a specific surface orientation, (*hkl*),
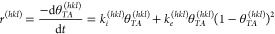
1

In a typical experiment, the Cu S^4^C surface starts with saturated monolayer coverage (θ_*TA*_^(*hkl*)^ = 1) at all surface orientations. During the initiation step, the initial vacancies needed for the explosion reaction are created. Once sufficient vacancy coverage is reached, kinetics of the explosion step dominate and determine the overall reaction rate until the TA is consumed. The increase in the vacancy coverage during decomposition causes the reaction rate to accelerate autocatalytically even under isothermal conditions.^22^ On the two enantiomers of each chiral surface structure, D-TA and L-TA, have different rate constants, leading to enantiospecific decomposition kinetics. For this work, the decomposition kinetics of TA enantiomers were measured on the three S^4^C samples centered on the (100), (110) and (111) directions. This data are used to model the enantiospecificity of the decomposition of TA on intrinsically chiral Cu surfaces.

Two approaches were used to model the enantiospecificity as a function of the surface structure and orientation. The first approach uses generalized coordination numbers (GCN) which have been found to be a useful surface structure descriptor for the reactivity of catalytic surfaces.^23,24^ The second approach uses a basis set expansion of cubic harmonic functions^25^ designed to capture the known symmetry of the surface structures around the low Miller index poles of the stereographic triangle. The two models were shown to fit the experimental data well. They were then used to make predictions about surface orientations that were not tested experimentally. The GCN model and the cubic harmonics model identified the surfaces with Miller indices of (11,3,1) and (17,5,2), respectively, to have the highest enantiospecificity. These two surface orientations are only 1.8° apart and thus can be tested experimentally using a single S^4^C which spans surface orientations that are up to 14 degrees apart. Moreover, the GCN model was used to identify which surface structure features are correlated with high enantiospecificity.

## 2 Methods

### 2.1 Experimental Data Set

#### 2.1.1 Data Collection

**Figure 2:**
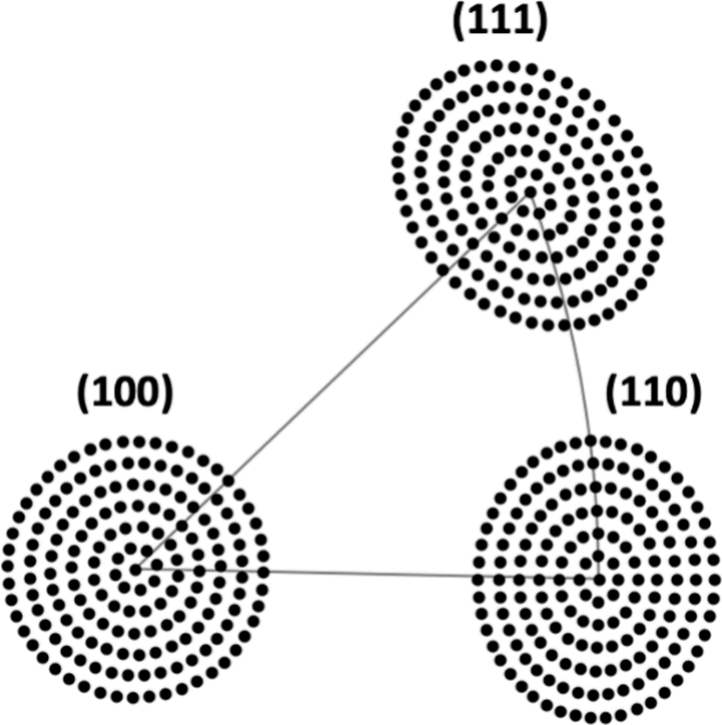
Equal area stereographic projection of the three S^4^C samples centered at the (100), (110), and (111) directions. The black dots represent the experimental sampling grid used for measurement of TA decomposition kinetics at 169 different surface orientations on each S^4^C sample.

The decomposition kinetics of D-TA and L-TA were measured on three Cu S^4^C samples centered on the (100), (110) and (111) directions.^21,26,27^ During isothermal heating at *T*_*iso*_ = 433 K, the temporal evolution of the local surface coverage, θ_*TA*_^(*hkl*)^(*t*; *T*_*iso*_) was measured experimentally for both TA enantiomers across a circular grid of 169 points on each S^4^C surface. Figure 2 shows equal-area projections of the 169 data collection points of the three S^4^Cs onto a 2D stereographic diagram viewed from the (100) direction. Although the equal area projection distorts the shapes of the S^4^C samples, we chose this projection to mitigate visual biases when comparing data clusters located in various regions of the sphere. The formula for calculating the equal-area projections is introduced in section S1 of the Supporting Information. This enables us to determine how much of the stereographic triangle is covered by the three S^4^C samples. A detailed discussion of the experimental protocol for data collection can be found in references^21,26,27^ though the following discussion will highlight and summarize the key findings required for this modeling study.

In a typical experiment, the S^4^C sample was first prepared by cleaning with cycles of Ar^+^ ion sputtering, followed by annealing at 900 K to restore the surface structure with confirmation by low energy electron diffraction (LEED). Second, a saturated TA monolayer was deposited onto the S^4^C surface by vapor phase exposure to the sample at 400 K to prevent multilayer formation. L-TA or D-TA monolayers were deposited from sublimation sources maintained at 390 K. Upon monolayer formation across the entire S^4^C surface, a series of heating cycles from 400 K to the isothermal reaction temperature of *T*_*iso*_ = 433 K were conducted to initiate TA decomposition at *T*_*iso*_ = 433 K for a short duration followed by quenching to 400 K to collect the data, θ_*TA*_^(*hkl*)^(*t*; *T*_*iso*_). Surface coverage was measured using spatially resolved X-ray photoemission spectroscopy (XPS) at 169 points on each sample surface. The surface coverage was quantified by measuring the O 1s signal intensity with XPS. After complete TA decomposition the halftime, *t*_1/2_^(*hkl*)^, or time at which the coverage reduces to half a monolayer is quantified at each sampling point. This experiment was repeated for the three S^4^C samples with both TA enantiomers.

Prior to modeling the surface enantiospecificity, the experimental data need to be aligned properly to directly map the decomposition halftimes to a surface orientation. To align the data, the physical sampling grid used for quantifying surface coverage through XPS must be converted to Miller indices so that the decomposition half-times can be directly mapped to a surface orientation. To convert the sampling grid points to Miller indices, electron backscatter diffraction (EBSD) was utilized to extract a pole figure for each S^4^C sample. The pole figures are used to determine the position and the orientation of the S^4^C samples in the stereographic triangle. The EBSD pole figures and full procedure of aligning the samples with the pole figures are shown in section S1 in the Supporting Information.

#### 2.1.2 Quantifying Surface Enantiospecificity

The TA coverage versus time data, θ_*TA*_^(*hkl*)^(*t*), was used to fit the rate law and obtain the rate constants, *k*_*i*_^(*hkl*)^ and *k*_*e*_^(*hkl*)^, on each surface denoted by its Miller index (*hkl*) for D-TA and L-TA separately. The decomposition half-time, *t*_1/2_^(*hkl*)^, is defined as the time to reach a half monolayer coverage. The *t*_1/2_^(*hkl*)^ can be measured directly or calculated using the two rate constants using the equation:
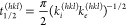
2The enantiospecificity is quantified by the difference in the half-times, Δ*t*_1/2_^(*hkl*)^, of the decomposition reactions of D-TA and L-TA enantiomers on each surface structure. The Δ*t*_1/2_^(*hkl*)^ is calculated based on the necessary diastereomeric relationship that was observed in these studies.^14,22,26,27^ This describes the relationship in which the decomposition halftime of L-TA on a surface with chirality S, *t*_1/2_^*L*/(*hkl*)–*S*^, should be equivalent to the halftime of D-TA on a surface with chirality R, *t*_1/2_^*D*/(*hkl*)–*R*^. Similarly, the halftime of D-TA on S, *t*_1/2_^*D*/(*hkl*)–*S*^, should be equivalent to that of L-TA on R, *t*_1/2_^*L*/(*hkl*)–*R*^. This relationship is captured in the halftime difference,
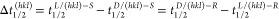
3The halftime difference is quantified on 169 surface structures on each of the three S^4^C samples, generating an experimental data set of 507 surface structures with their corresponding enantiospecificity quantified by the halftime difference. Using this data set, we can build quantitative models of the enantiospecificity as a function of the surface orientation and structure.

### 2.2 Modeling Approaches

We developed two approaches to modeling the surface enantiospecificity of TA decomposition kinetics as a function of the surface structure. The first approach uses generalized coordination numbers (GCN) to relate the surface structure to the enantiospecificity. The second approach uses a series expansion of cubic harmonic functions to approximate the half-time difference function.

#### 2.2.1 Generalized Coordination Numbers (GCN) Model

An approach to modeling surface structure dependent reaction kinetics is to use descriptors that capture the relationship between the surface structure and its reactivity. An activity descriptor that captures this relationship is the coordination number (CN). Each surface is composed of atoms at which reactions occur. CN is calculated by counting the number of nearest neighbor atoms of each surface atom. An atom with a lower CN is more reactive than an atom with higher CN.^28^ Thus, a surface with a high density of low coordination atoms is more reactive than a surface with a lower density of such atoms. The Generalized Coordination Number (GCN) is an extension of the CN which was found to be a better chemical activity descriptor for surface reactions.^29,30^ GCN for a given surface atom is the sum of the CNs of the surrounding atoms normalized by the maximum CN value. Thus, GCN combines information about the first and second neighbors of each atom. This allows it to capture more information about the local structure and captures the structure sensitivity more accurately. Many studies have shown that GCN is a good activity descriptor for modeling monometallic and alloy surfaces.^23,24^ Therefore, in this study, GCN features will be used to build a model to understand and predict the enantiospecific decomposition of TA on Cu(*hkl*) surfaces of arbitrary orientation.

**Figure 3:**
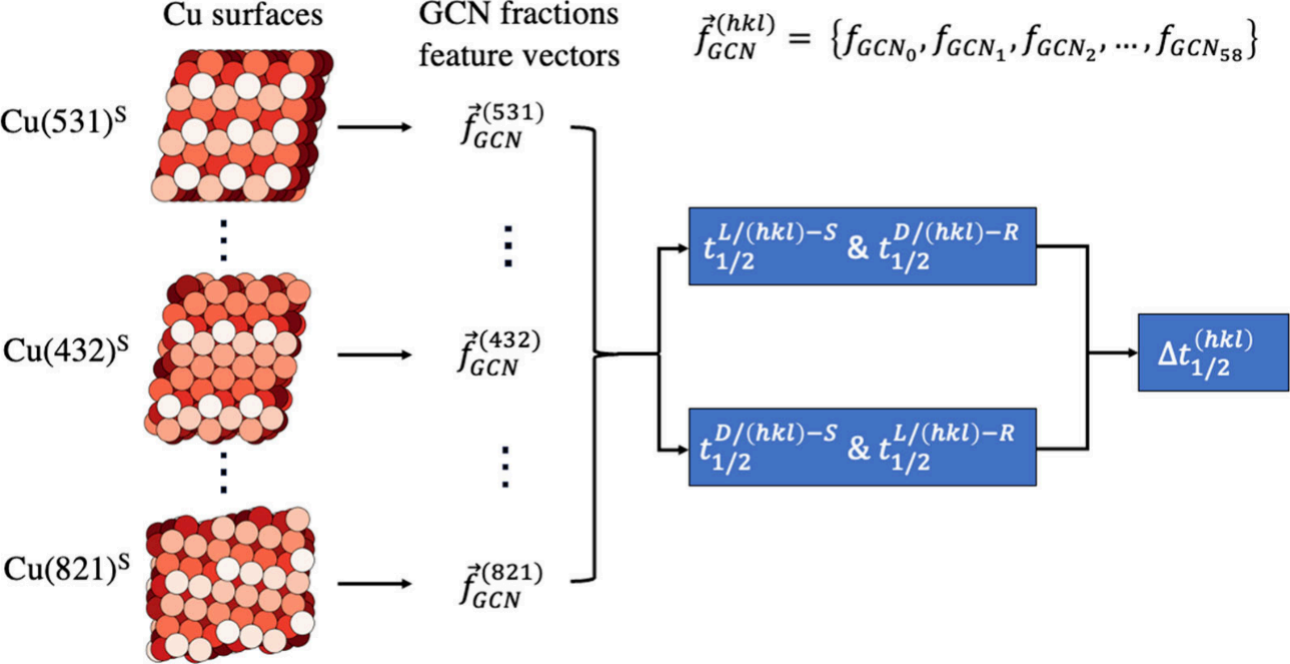
An illustration of the method for predicting the half-time difference using the GCN model. The Cu atoms are colored based on the GCN values where lighter colors represent lower GCN values. Each surface orientation is represented by a vector that contains the fraction of surface atoms with each of the 58 unique GCN values. The feature vectors are used to predict the decomposition halftime based on a diastereomeric relationship between the surface and the adsorbate chirality. The predicted halftime difference is computed by subtracting the halftimes of D- and L-TA.

Since there is an infinite number of surface orientations, we computed the GCN features of 270 surfaces that span the whole stereographic triangle, as shown in Figure S3. We then used linear interpolation to calculate the GCN features for the experimental surface orientations. Each of the 270 surface orientations was computationally represented as a collection of atoms arranged in a way to reflect its corresponding surface structure. The Slab Generator class in the Pymatgen^31^ library was used to construct these representations. To calculate the GCN feature representation of each surface, the GCN values for each atom on the surface are calculated by summing the CNs of the surrounding atoms normalized by the maximum CN value on that surface. It was found that there are 58 unique GCN values across all of the surface orientations. Therefore, each surface is represented by a 58-long feature vector that is computed by counting the number atoms with each GCN value and dividing by the number of surface atoms as shown in Figure 3. The surface atoms are defined as atoms that have a CN less than 12 which is the highest CN of atoms in an FCC structure. A linear regression model was used to quantify the relationship between these GCN feature vectors and their corresponding decomposition half-time values, *t*_1/2_, for a given enantiomer of TA.

Generalized coordination numbers capture information about the surface structure but do not capture information about the handedness R(S) of the surface. For instance, the GCN feature representation of Cu(531)-R is identical to that of Cu(531)-S. Therefore, it is necessary to provide the model with information about the handedness. One way to achieve that is by splitting the data into two data sets based on the diastereomeric relationship described in eq 3. The data set was split into a first data set that has the L-TA decomposition halftimes on S surfaces and D-TA halftimes on R surfaces since the relationship suggests that *t*_1/2_^*L*/(*hkl*)–*S*^ should be equivalent to *t*_1/2_^*D*/(*hkl*)–*R*^. The second data set has D-TA halftimes on S surfaces and L-TA halftimes on R since the relationship also mandates that *t*_1/2_^*D*/(*hkl*)–*S*^ should be equivalent to *t*_1/2_^*L*/(*hkl*)–*R*^. Two separate GCN models were trained on the two data sets and are used for predicting the D-TA and L-TA halftimes separately. The predicted halftime difference is quantified by the difference in the predicted halftimes on each surface. Figure 3 illustrates the method of predicting the half-time difference using the GCN model. Besides making predictions about the halftime difference on a specific surface orientation, this model can provide information about the specific surface structure features that make one surface structure more enantiospecific than another.

#### 2.2.2 Cubic Harmonics Model

**Figure 4:**
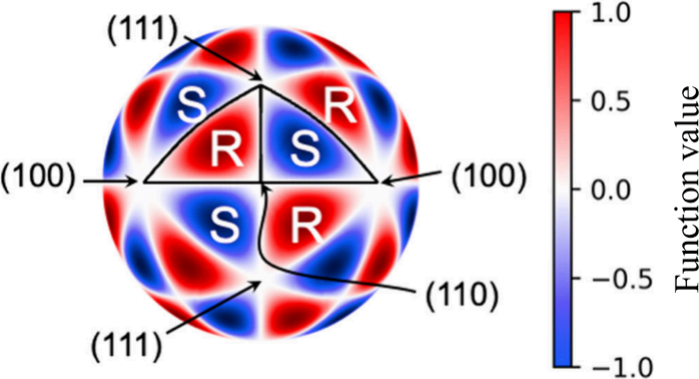
A visualization of the ninth degree cubic harmonic function that has 4-fold, 3-fold, and 2-fold rotational symmetry around the (100), (111) and (110) orientations, resembling the symmetry of the FCC Cu crystal. The values of the function over the surface of the sphere are normalized between −1 and 1 as shown in the color bar. The alternation of the positive and negative values resembles the alternation of the R(S) handedness on the FCC Cu crystal.

The halftime difference function, Δ*t*_1/2_^(*hkl*)^(θ, ϕ), is defined on the surface of a spherical Cu crystal where different orientations, (θ, ϕ), correspond to different surface structures. Spherical harmonics form an orthogonal basis set that can approximate any square-integrable function defined on the surface of a sphere; they are spherical analogs of the sines and cosines that allow a Fourier series expansion to express functions in flat space. While the half-time difference function can be approximated by an expansion using a basis set of spherical harmonics, the symmetry of the face-centered cubic (FCC) lattice imposes cubic symmetry. Only linear combinations of spherical harmonics with the appropriate cubic symmetry, referred to as cubic harmonics,^25^ may contribute to the expansion. These specific spherical harmonics must be antisymmetric with respect to inversion and reflection, and thus, we call them chiral cubic harmonics. Figure 4 shows the ninth degree cubic harmonic function, which is the simplest of the spherical harmonic functions that satisfy both cubic symmetry and inversion antisymmetry. The function has 4-fold, 3-fold, and 2-fold symmetry along the labeled (100), (111), and (110) axes. It has antisymmetric mirror planes reflected by the alternation between positive (red) and negative (blue) values which resembles the alternation of the R(S) handedness on the FCC Cu crystal. A linear combination of five chiral cubic harmonic functions was used to model the halftime difference function. The full mathematical procedure to obtain these chiral cubic harmonic functions is provided in section S4 of the Supporting Information.

### 2.3 Model Evaluation

To evaluate the accuracy of the two models, the data were subdivided randomly into 80% for training (fitting) and 20% for testing. Linear regression models with Ridge regularization and L2 regularization were found to fit well, improve numerical stability, and reduce the risk of overfitting. The Ridge regularization strength hyperparameter, alpha, was determined using a grid search cross validation method, GridSearchCV, from the Scikit-learn^32^ library. The grid search was done on alpha values between 0.001 and 1. The best alpha values for the GCN model and the chiral cubic harmonics model were found to be 0.01 and 0.12, respectively. The mean absolute errors and the *R*^2^ scores evaluation metrics are reported in the Results and Discussion section. After evaluating the ability of the models to fit the available experimental data, we then use them to inform efforts to experimentally map TA decomposition in as yet unexplored regions of the surface orientation space. Additional experiments should focus on the surface structures with the highest predicted enantiospecificity. To determine the surface with the highest enantiospecificity, we used the models to make extrapolative predictions using a grid of surfaces that spans the whole stereographic triangle. The limitations of the machine learning methods in extrapolating beyond the training data are well-known. Therefore, we will rely on future experiments to test the models’ ability to identify the surface orientation with the highest enantiospecificity. The code to fit and evaluate these models can be accessed through https://doi.org/10.5281/zenodo.13257089.

## 3 Results and Discussion

### 3.1 Experimental Data Set

**Figure 5:**
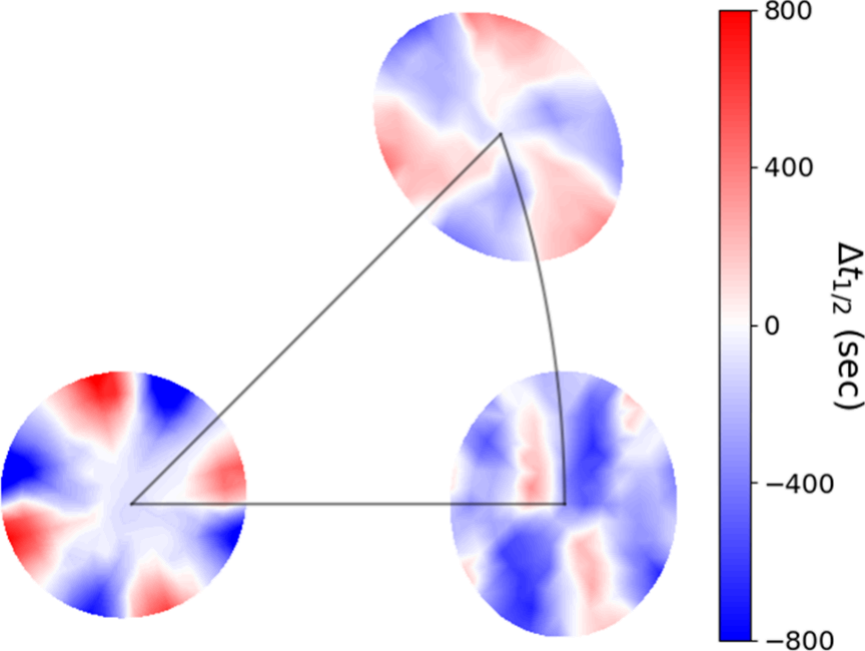
Mapping of the halftime difference, Δ*t*_1/2_^(*hkl*)^, data collected on the (100), (110), and (111) samples which have 4-fold, 2-fold, and 3-fold rotational symmetry, respectively. The red regions of each sample contain surfaces where the D-TA halftime is larger than that of L-TA. The blue regions contain surfaces where the L-TA halftime is larger than the D-TA halftime. The half-time difference should be zero (white) along the high symmetry directions.

The experimental halftime difference, Δ*t*_1/2_^(*hkl*)^, data calculated using eq 3 is shown in Figure 5. The red regions of each sample contain surfaces where the D-TA half-time is larger than that of L-TA. The blue regions contain surfaces where the L-TA half-time is larger than the D-TA halftime. The half-time difference should be zero (white color) along the high symmetry directions. The more saturated the color, the higher the enantiospecificity. Due to the symmetry of the FCC Cu crystal, the Cu(100), Cu(110), and Cu(111) S^4^C samples have 4-fold, 2-fold, and 3-fold rotational symmetry, respectively. The alternation of the sign results from the alternating handedness R(S) of the surfaces in different regions of each sample. Surfaces with R-handedness do not always have a positive halftime difference, and vice versa for the surfaces with S-handedness. This shows that knowing only the surface handedness, one cannot determine whether D-TA or L-TA would have a larger halftime. Rather, both the specific surface structure and the orientation are what determine the sign and the magnitude of the halftime difference. One challenge in modeling these data is capturing the symmetry and the sign of the halftime difference values in the different surface regions on each S^4^C. The experimental error present in the data poses another challenge to modeling.

### 3.2 Model Evaluation

**Figure 6:**
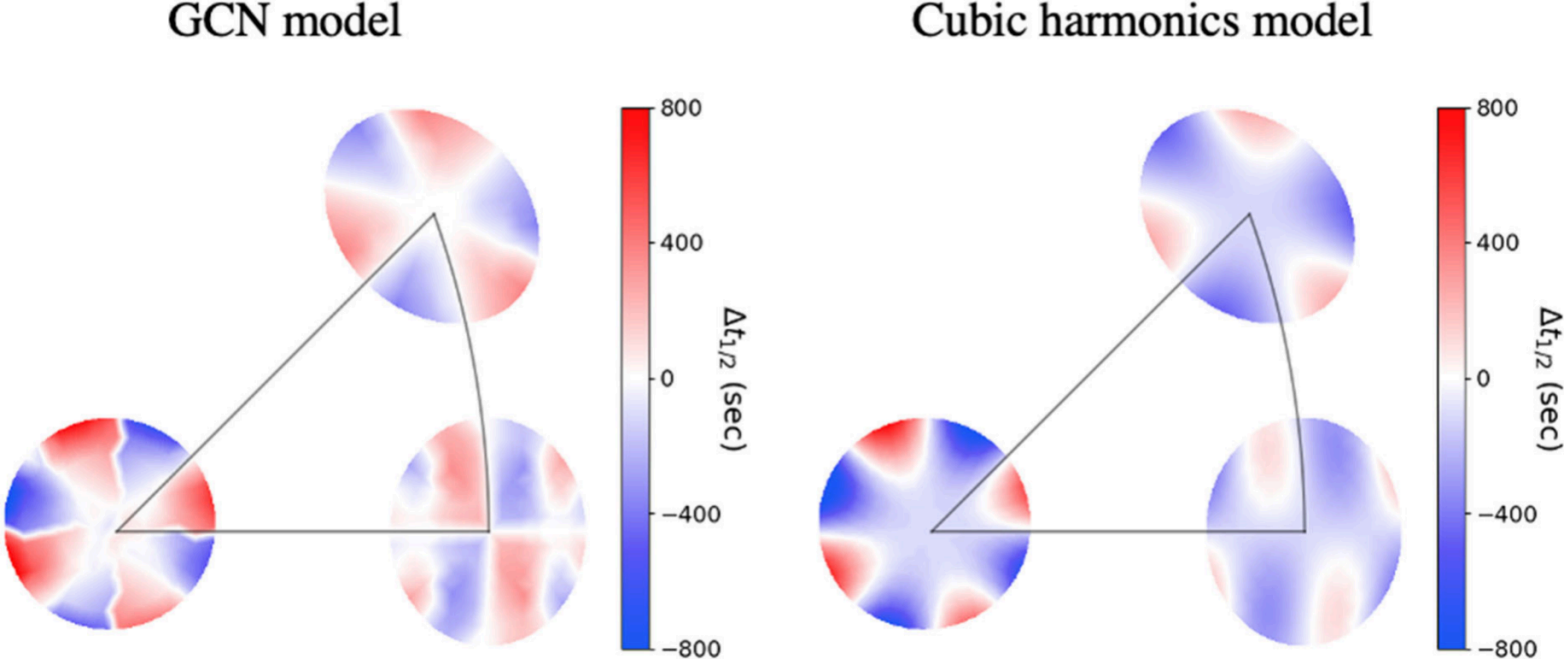
Visualization of the predictions of the GCN and the cubic harmonics models. Both models fit the symmetry and the orientation of the experimental data.

The two models were evaluated both quantitatively and qualitatively. Quantitatively, the GCN model has a mean absolute error, MAE, of 141 s for the training set and 138 s for the test set. The cubic harmonics model has lower errors with an MAE of 129 s for the training set and 133 s for the test set. The similarity of between the MAEs of the training and test set of both models indicates a low risk of overfitting to the training data. The test parity plots of the two models are shown in section S5 of the Supporting Information. Although the cubic harmonics model has only 5 features and the GCN model has 58 features, the chiral cubic harmonics model performs better quantitatively. Qualitatively, Figure 6 shows that the predictions of the two models reflect the symmetry and the orientation of experimental data that belong to each S^4^C. The (100), (110), and (111) predictions have 4-fold, 2-fold, and 3-fold symmetry, respectively. They are also correctly oriented such that the red regions of the (100) and (110) as well as the blue region of the (111) sample are inside the triangle. The two models fit the available experimental data well based on the MAEs and the visualization of the predictions.

### 3.3 Identification of the Most Enantiospecific Surface Orientation

**Figure 7:**
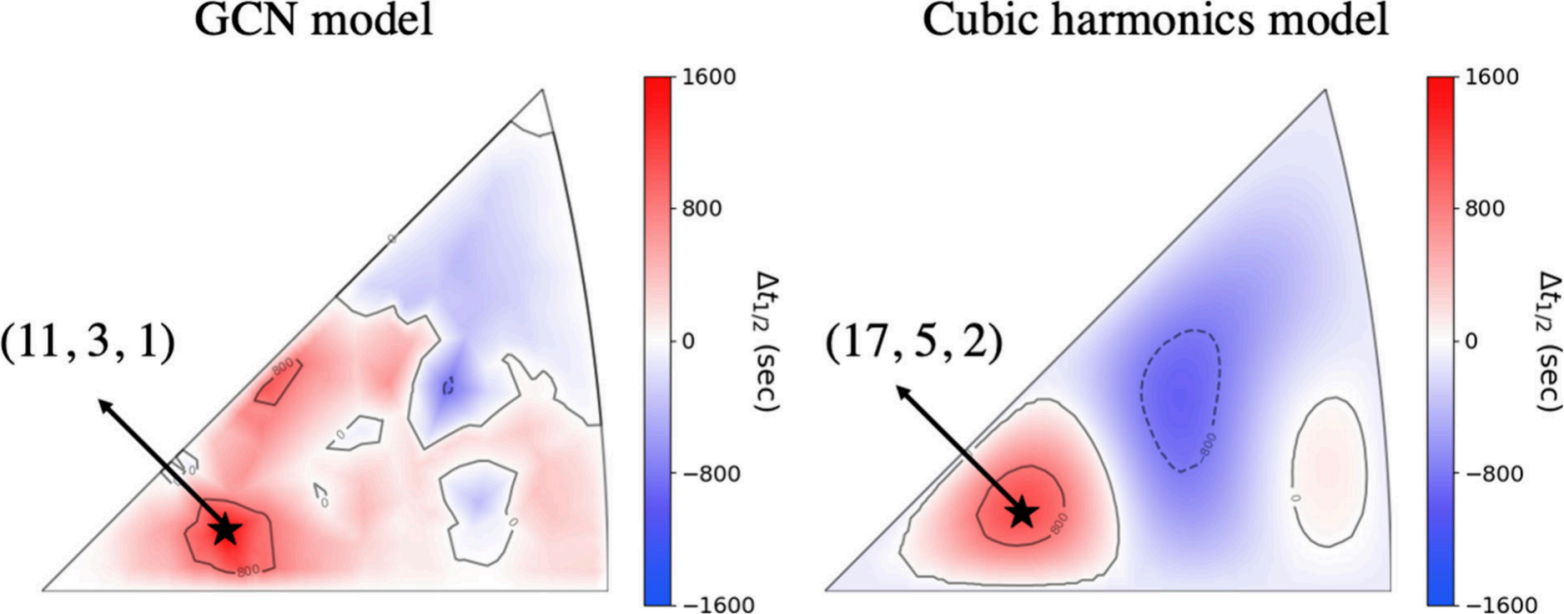
Contour plots of the halftime difference models’ predictions of a grid of surface orientations that span the stereographic triangle. The GCN and the harmonics models predict (11,3,1) and (17,5,2) respectively to have the highest enantiospecificity.

After the two models were fitted to the experimental data, they were used to make predictions about a grid of surface orientations that spans the stereographic triangle. The two models were used to predict the halftime difference of TA decomposition on a grid of surface orientations with Miller indices up to *h* = *k* = *l* = 30 that span the stereographic triangle. Figure 7 shows the contour plots of the two models’ predictions, where the red color represents surfaces with positive half-time difference and blue points represent the surface orientations with negative Δ*t*_1/2_^(*hkl*)^. The contour plot of the cubic harmonic model is smoother than the GCN model contour plot. This is because the individual GCN features are nonsmooth functions of the surface orientations while the individual cubic harmonic functions are smooth functions as shown in section S6 in the Supporting Information. Moreover, unlike the GCN features, the cubic harmonic functions are smooth functions of the surface orientations. The GCN model predicted that the surface with the Miller index (11,3,1) is the most enantiospecific surface. The chiral cubic harmonic model predicted the (17,5,2) surface to be the most enantiospecific. We found that if we train the GCN model or the chiral cubic harmonics model on different random 80% of the data, the magnitude of the extrapolated halftime predictions will vary. However, the location of the surface with the highest enantiospecificity predicted by each model was found to be consistent. Section S7 in the Supporting Information shows this result with more details. Although the two models predict two different surface orientations with the highest enantiospecificity, the two models agree that the surfaces that lie in the left-hand corner of the triangles have high enantiospecificity. The two optimal surface orientations are only 1.8 degrees apart. Therefore, they can be experimentally tested using a single S^4^C sample which can be used to test surfaces that are oriented up to 14 degrees apart. Having two different models, the GCN and the harmonic models, agree on the location of the next experiment gives us more confidence in the predictions. However, testing these predictions experimentally is the only method to verify the accuracy of these predictions.

**Figure 8:**
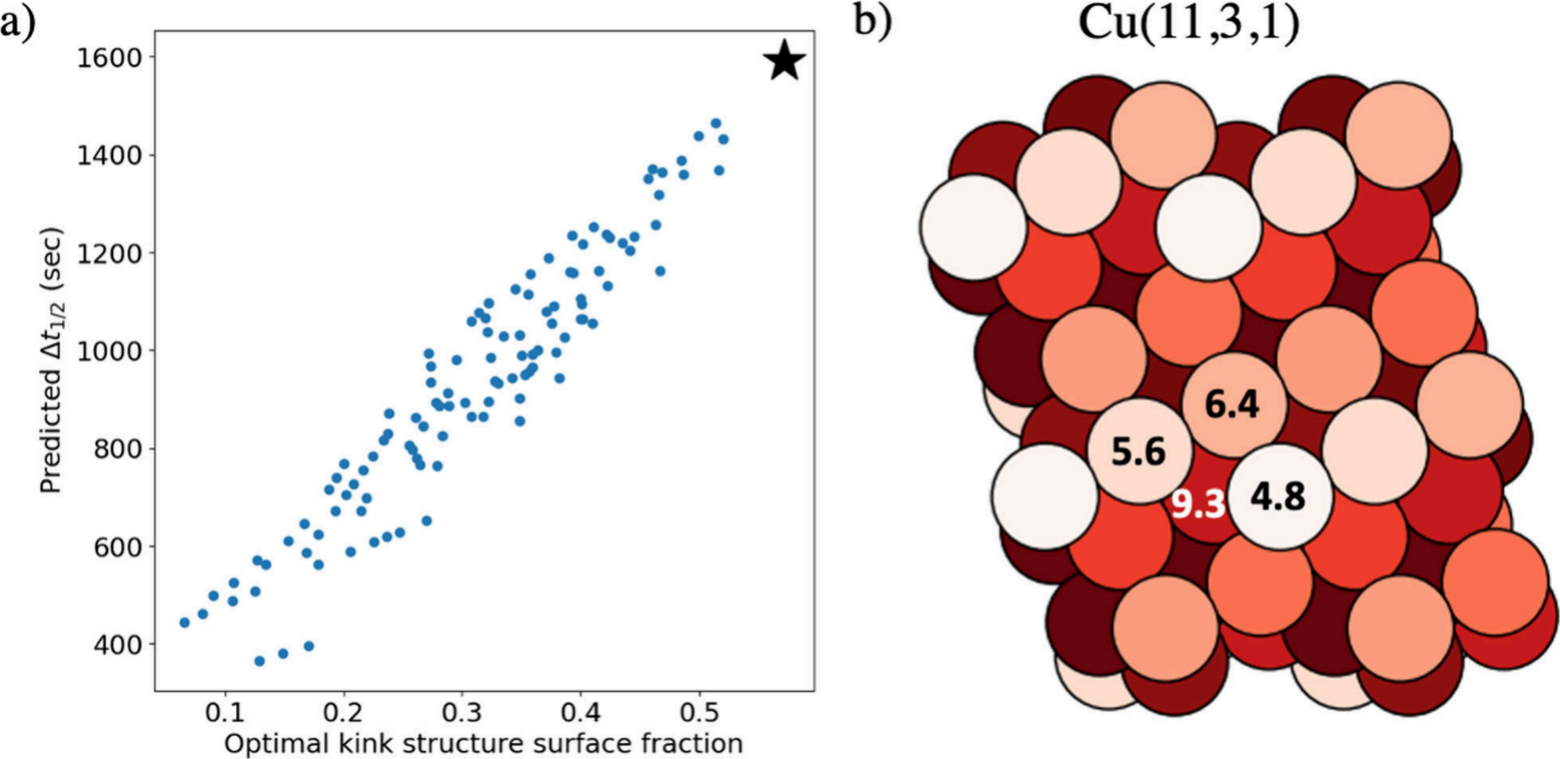
(a) Trend between the fraction of atoms on the surface that form the optimal kink structure which is composed of atoms with GCN values of 4.8, 5.6, 6.4, and 9.3 and the corresponding predicted halftime difference. The black star is the (11,3,1) predicted to have the highest enantiospecificity by the GCN model. (b) A representation of the Cu(11, 3, 1) with atoms colored based on the GCN values where lighter colors represent lower GCN values. The GCN values of the atoms that form the optimal kink structure are highlighted.

Although the cubic harmonic model is simpler than the GCN model since it has a lower number of features, the GCN model is more interpretable since the features that are used capture physical details about the surface structure. Therefore, the GCN model can be used to infer what makes (11,3,1) the most enantiospecific surface orientation. We found that there is a specific kink structure composed of atoms with GCN values of 4.8, 5.6, 6.4, and 9.3 that is correlated with increasing the halftime difference prediction. As shown in Figure 8, surfaces with a higher fraction of atoms that form this optimal kink structure have a higher predicted halftime difference, and thus are more enantiospecific. The (11,3,1) surface orientation, highlighted by the black star, was predicted to have the highest enantiospecificity, because it has the highest fraction of atoms that form the optimal kink structure. It is important to mention that the decomposition of tartaric acid can cause surface reconstructions similar to what was shown experimentally by Reinicker et al. for aspartic acid decomposition on Cu.^33^ These reconstructions could disrupt the kink structure identified here, affecting the trend in the predicted halftime difference.

Studying the effect of surface reconstructions on the kinetics is a very daunting and computationally expensive task^34^ that was not investigated in this work. The GCN model relies on the assumption that the reconstructed surface should be dependent on the structure of the surface before reconstruction. The cubic harmonic model, on the other hand, does not make any assumptions about surface atomic structure. The agreement between the two models on the surface with the maximum enantiospecificity shows that the GCN model could still describe the surface chirality even without accounting for surface reconstructions. Therefore, the results of the GCN model can still be useful in designing experiments studying the enantiospecificity of chiral adsorbates on chiral surfaces.

## 4 Conclusions

The surface structure sensitivity of the enantiospecific behavior of the TA vacancy-mediated decomposition reaction as a function of the chiral copper surface orientations was modeled using two different approaches. The first approach employed physics-informed features known as generalized coordination numbers (GCN) to establish a correlation between the surface structure and enantiospecificity. The second approach employed mathematical symmetry features, utilizing a basis set of chiral cubic harmonics to approximate the enantiospecific behavior. The two models’ predictions display important features of the data, such as having the correct symmetry and the orientation of the data that belong to each S^4^C. The GCN model and cubic harmonics model predicted the (11,3,1) and (17,5,2) surface orientations respectively to have the highest enantiospecificity. Because of the proximity of these two surface orientations, they can be tested experimentally using a single S^4^C to determine the accuracy of the models’ predictions. The GCN model was used to identify a specific kink structure composed of atoms with GCN values of 4.8, 5.6, 6.4, and 9.3 that is correlated with high enantiospecificity. The (11,3,1) was found to be optimal because it has the highest fraction of atoms that form this kink structure. The modeling framework developed in this work will be useful in minimizing the experimental data needed to predict the behavior of different chiral molecules reacting on chiral surfaces.

## Supporting Information Available

Additional experimental and mathematical and computational details (PDF)
