# Supplementary material for: Structure Sensitive Reaction Kinetics of Chiral Molecules on Intrinsically Chiral Surfaces
Source: J Phys Chem C Nanomater Interfaces. 2024 Aug 13;128(33):13879–87. doi: 10.1021/acs.jpcc.4c04224 (PMC11345842; doi:10.1021/acs.jpcc.4c04224)
Supplement: Supplementary file 1 — jp4c04224_si_001.pdf [file jp4c04224_si_001.pdf]

Supplementary Information:

Structure Sensitive Reaction Kinetics of Chiral Molecules on Intrinsically Chiral Surfaces

[1]Kareem Abdelmaqsoud, [1]Michael Radetic, [1]Carlos Fernández-Cabán,[2]Michael Widom, [1]John R. Kitchin, [1]Andrew J. Gellman

[1]Department of Chemical Engineering

[2]Department of Physics

Carnegie Mellon University

5000 Forbes Ave, Pittsburgh, PA 15213

Contents:

1- Equal area stereographic projection
2- Conversion of S^4C sample coordinates to miller indices
3- Computational details of the Generalized Coordination Numbers (GCN) model
4- Mathematical details of the cubic harmonic functions
5- Test parity plots.
6- The GCN features are non-smooth functions of the surface orientations unlike the cubic harmonics functions.
7- Effect of changing the training data on the extrapolation of the models

1. Equal area stereographic projection

Various types of stereographic projections could be used to project the Miller indices of the different surface orientations into a 2D plane on the stereographic triangle. We chose the equal-area stereographic projection because it helps mitigate visual biases when comparing data clusters located in various regions of the sphere. The equal-area projection introduces distortion to geometric shapes like small circles that are not centered at the sphere's pole. This distortion effect is highlighted in figure 2 in the main paper where points on the Cu(111) sample do not form circular grid while the points on Cu(100) sample are not distorted because they are centered at the sphere pole. Despite this distortion, a constant area on the projection corresponds to a constant area on the sphere's surface, irrespective of its position. Equation (1) shows the formula to calculate the projected X, Y coordinates using the Miller indices (hkl) that represent each surface orientation.

$$(X, Y) = \left(k\sqrt{\frac{2}{1+h}}, l\sqrt{\frac{2}{1+h}} \right) \tag{1}$$

2. Conversion of S^4C sample coordinates to Miller indices

Prior to modeling the surface structure sensitivity of TA decomposition on Cu, the physical sampling grid used for quantifying surface coverage through XPS must be converted to Miller indices. This is necessary to directly map the decomposition halftimes to a surface orientation. First, the circular sampling grid is converted to three-dimensional vectors provided that the topmost surface is spherically polished with a 21 mm radius of curvature. Second, to achieve the conversion from these three-dimensional vectors to Miller indices, electron backscatter diffraction (EBSD) was utilized to extract a pole figure for each S^4C sample. EBSD can be used to identify the pole positions and surface orientation of the S^4C samples by defining the basis vectors at which the data is obtained experimentally. Figure (S1a) shows the stereographic projection along the (100) direction. Figure (S1b) shows the EBSD pole figure of the Cu(100) S^4C sample along the (100) direction. Comparing the two figures shows that the experimental S^4C sample is well centered or in other words the normal of the sample is in fact (100). However, slight in-plane offset between the EBSD pole figures and our stereographic projection. For instance, the (001) pole position is offset by ~14.5° from the vertical coordinate. As a result, $(\boldsymbol{a}, \boldsymbol{b}, \boldsymbol{c})$ basis vectors for our sample

would be $\boldsymbol{a} = [0,0.97,0.25]$, $\boldsymbol{b} = [0,-0.25,0.97]$, and $\boldsymbol{c} = [1,0,0]$. Figure (S2) shows the EBSD pole figures for the Cu(100), Cu(110) and Cu(111) along the (100), (110) and (111) directions. Dr. Michael Radetic's dissertation[1] describes in detail the procedure of obtaining the basis vectors of the other S^4C samples.

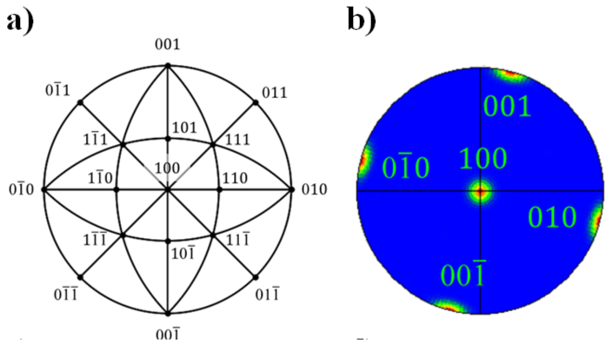

Figure S1: Pole figure diagrams providing information on surface crystallinity and surface orientation. In the first image a) a 100 stereographic projection serves as a useful reference for identifying pole positions in a diffraction pole diagram using a technique such as EBSD. Image b) represents the pole diagram for the Cu(100) S^4C along the (100) direction.

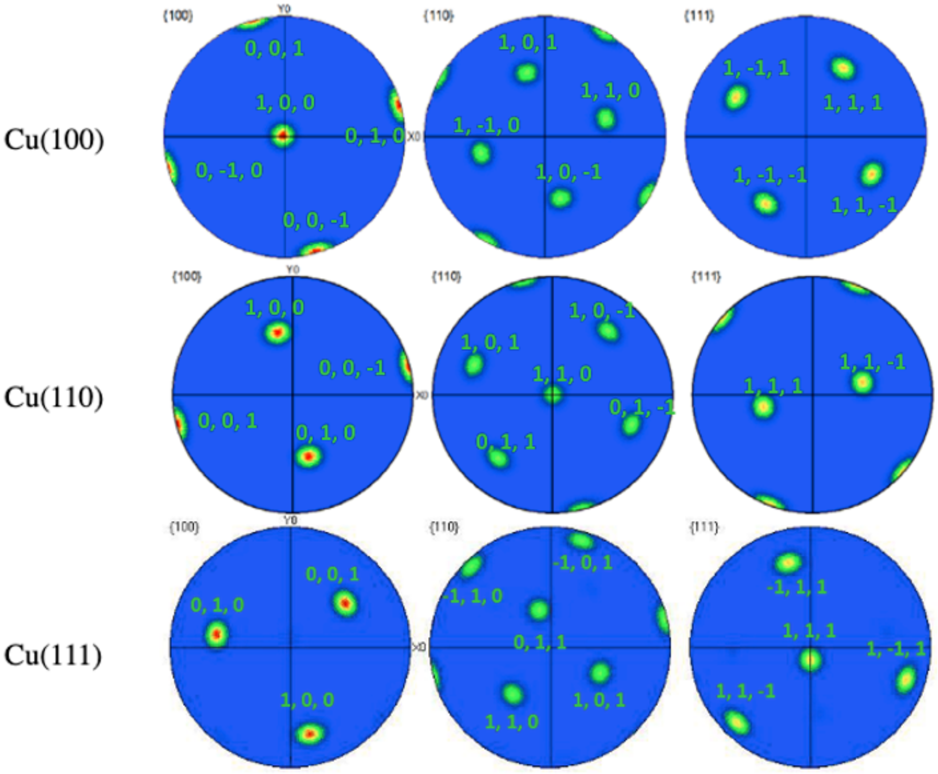

Figure S2: the EBSD pole figure diagrams for the Cu(100), Cu(110) and Cu(111) S^4C samples.

3. Computational details of the Generalized Coordination Numbers (GCN) model

To build the GCN model, we randomly selected surface orientations that span the stereographic triangle and calculated the GCN feature vector of each surface orientation. Figure S3 shows the locations on the stereographic triangle of the 270 surface orientations selected randomly. We used the LinearNDInterpolator method from the SciPy[2] library to calculate GCN feature vectors of all surface orientations needed for this study. The linear interpolation was done in 2D based on the projected x and y coordinates of each surface orientation in the stereographic triangle. After computing the GCN feature vectors, the R(S) handedness of each surface orientation is determined to split the dataset into $t_{1/2}^{L/(hkl)-S}$ and $t_{1/2}^{D/(hkl)-R}$ dataset and $t_{1/2}^{D/(hkl)-S}$ and $t_{1/2}^{L/(hkl)-R}$ dataset. First, all achiral surface structures with $h = k = l = h$ or $h \times k \times l = 0$ are removed from the experimental dataset and a value of zero is assigned to halftime difference. Second, the Attard et al.[3] notation which assigns an R or S designation to any kinked FCC surface was used to determine the handedness R(S) based on the Miller indices of each surface orientation.

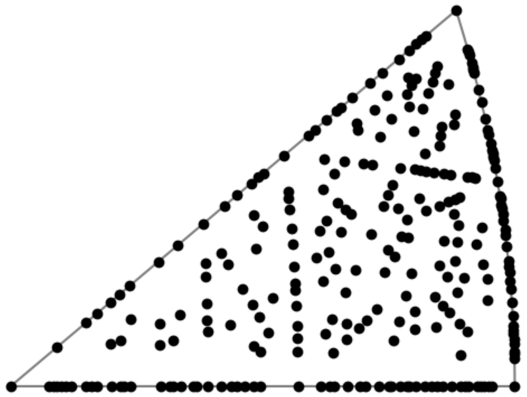

Figure S3: Stereographic projection of the surface orientations that were selected randomly for interpolation.

4. Mathematical details of the cubic harmonic functions

The halftime difference $\Delta t_{1/2}^{(hkl)}(\theta,\phi)$ can be modelled directly using spherical harmonics (Y_l^m), which are an orthogonal basis set that can approximate any square-integrable function on the surface of a sphere. Spherical harmonics are the spherical analogs of the sines and cosines that allow a Fourier transform to approximate any function in flat space. Because the surfaces are invariant under rotations belonging to the cubic symmetry group G, only certain linear combinations of (Y_l^m) are allowed, which known as cubic harmonics. Group representation theory allows us to identify these functions.

Specifically, for each value of the total angular momentum l we seek combinations of Y_l^m that transform as the identity representation. The number of independent occurrences of representation α in angular momentum l is

$$n_l^{(\alpha)} = \frac{1}{|G|}\sum_{g\in G}\chi_\alpha^*(g)\chi_l(g), \tag{2}$$

where $|G| = 24$ for the cubic rotation group, $\chi_0(g) = 1$ for the unit representation $\alpha = 0$, and the character for a rotation angle θ under angular momentum l is:

$$\chi_l(\theta) = \frac{\sin[(l+1/2)\theta]}{\sin[\theta/2]}. \tag{3}$$

Application of this formula yields $n_l^0 \geq 1$ for even values of $l = 0,4,6,8,10,\ldots$ and for odd values of $l = 9,13,15,17,19,\ldots$. Because $f(\theta,\phi)$ is odd under inversion, only odd l are allowed in the expansion of $\Delta t_{1/2}^{(hkl)}$. In some cases, multiple independent instances of the identity are contained in a given angular momentum, for example $n_l^0 = 2$ for $l = 12$ and for $l = 21$. To produce a cubic harmonic $K_l(\theta,\phi)$, we apply a projection method. Specifically, we start with a spherical harmonic $Y_l^m$and superpose its rotated variants for all elements of G,

$$K_l(\theta,\phi) = A\sum_{g\in G}\sum_{m'=-1}^{l} Y_l^m(\theta,\phi)D_{m',m}^l(g) \tag{4}$$

with $D_{m',m}^l(g)$ the Wigner D-matrix, and A a normalization factor. By construction, K_l^m is invariant under all cubic rotations, however it usually vanishes identically. Nonvanishing results are obtained for the values of lm such that $n_l^0 \geq 1$and m is a multiple of 4. The first five functions for odd l are shown in table S1. The functions are normalized over the surface of a sphere such

that <f | f>=1. The functions are provided in cartesian coordinates and can be converted to spherical coordinates using the following transformations:

$$x = \sin(\theta)\cos(\phi);\ y = \sin(\theta)\sin(\phi);\ z = \cos(\theta) \tag{5}$$

Table S1: mathematical expressions of the five cubic harmonic functions used in the cubic harmonics model in cartesian coordinates. All the functions are normalized over the surface of the sphere. A represents the normalization factor multiplied by each function.

Cubic harmonic functions	A
$K_9^4(x,y,z) = A\,xyz(x^2-y^2)(y^2-z^2)(z^2-x^2)$	$\frac{1}{8}\sqrt{\frac{4849845}{2\pi}}$
$K_{13}^4(x,y,z) = A\,xyz(x^2-y^2)(x^2-z^2)(y^2-z^2)(378+575x^4 + 575y^4 - 828z^2 + 575z^4 + 23y^2(-36+25z^2) + 23x^2(-36+25y^2+25z^2))$	$\frac{3}{160}\sqrt{\frac{323323}{2\pi}}$
$K_{15}^4(x,y,z) = A\,xyz(x^2-y^2)(x^2-z^2)(y^2-z^2)(-154+435x^6 + 435y^6 + 550z^2 - 825z^4 + 435z^6 + 15y^4(-55 + 29z^2) + 15x^4(-55+29y^2+29z^2) + 5y^2(110 - 165z^2 + 87z^4) + 5x^2(110+87y^4-165z^2+87z^4 + 3y^2(-55+29z^2)))$	$\frac{1}{64}\sqrt{\frac{104786045}{2\pi}}$
$K_{17}^4(x,y,z) = A\,xyz(5005x^4(y^2-z^2) - 25740x^6(y^2-z^2) + 62205x^8(y^2-z^2) - 70122x^{10}(y^2-z^2) + 29667x^{12}(y^2-z^2) + y^2z^2(5005y^2 - 25740y^4 + 62205y^6 - 70122y^8 + 29667y^{10} + z^2(-5005 + 25740z^2 - 62205z^4 + 70122z^6 - 29667z^8)) + x^2(-5005y^4 + 25740y^6 - 62205y^8 + 70122y^{10} - 29667y^{12} + z^4(5005 - 25740z^2 + 62205z^4 - 70122z^6 + 29667z^8)))$	$\frac{5}{128}\sqrt{\frac{156009}{29\pi}}$

$K_{19}^{4}(x,y,z) = A\,xyz(-2457x^4(y^2-z^2) + 16965x^6(y^2-z^2) - 58435x^8(y^2-z^2) + 105183x^{10}(y^2-z^2) - 94395x^{12}(y^2-z^2) + 33263x^{14}(y^2-z^2) + y^2z^2(-2457y^2 + 16965y^4 - 58435y^6 + 105183y^8 - 94395y^{10} + 33263y^{12} + z^2(2457 - 16965z^2 + 58435z^4 - 105183z^6 + 94395z^8 - 33263z^{10})) + x^2(2457y^4 - 16965y^6 + 58435y^8 - 105183y^{10} + 94395y^{12} - 33263y^{14} + z^4(-2457 + 16965z^2 - 58435z^4 + 105183z^6 - 94395z^8 + 33263z^{10})))$	$\frac{15}{256}\sqrt{\frac{7436429}{62\pi}}$

Figure S3 shows a visualization of the five functions used in the cubic harmonics model, $l = 9,13,15,17,19$, which all have the cubic symmetry elements. These functions differ in the frequency at which the sign of the function changes on the surface of the sphere. A higher degree function has a higher frequency of sign changes. We did not include cubic harmonic functions with orders higher than L=19 because they were found to overfit to the training data and produce unrealistic extrapolative predictions. Before being used to fit a linear regression method, these functions were normalized over the surface of the sphere. Linear regression was used to find the coefficients that we multiply by these functions to minimize the difference between the model predictions and the experimental data.

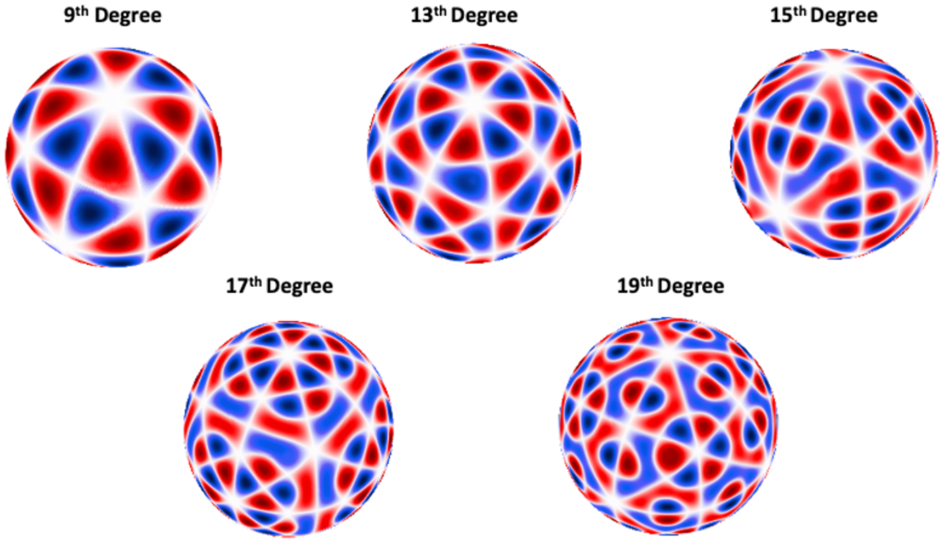

Figure S4: A visualization of the five cubic harmonic functions used in the cubic harmonics model. All the functions have the same cubic symmetry elements, but the frequency of the positive/negative signs increase as the degree of the cubic harmonic function increases.

5. Test parity plots

Although the cubic harmonics model is a simpler model than the GCN model since it has only 10 features compared the GCN model which has 58 features, the cubic harmonics model fits the data better. Figure S5 shows a comparison of the test parity plots of the GCN and the cubic harmonics models. The GCN model has a test R^2 value, coefficient of determination, of 0.57 while the cubic harmonics model has a test R^2 value of 0.72. The test R^2 values are similar to the training R^2 values which are 0.64 and 0.66 for the GCN and the cubic harmonics models respectively. Therefore, the models show no clear signs of overfitting or underfitting to the experimental data.

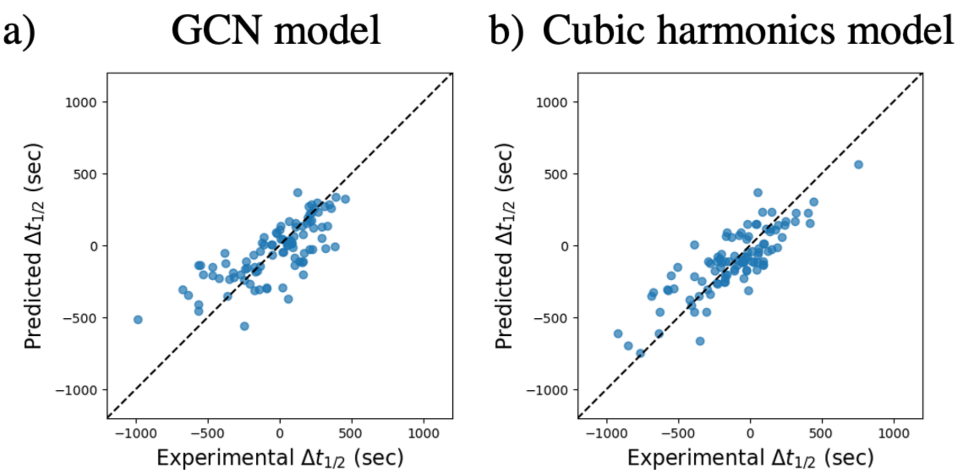

Figure S5: Test parity plots of the GCN and the cubic harmonics models. The two models fit the data with errors that are within the experimental errors.

6. The smoothness of the GCN features and the cubic harmonic functions

It was shown in figure 9 that the contour plot of the cubic harmonics model predictions is smoother than the GCN model plot. This is because the individual GCN features are non-smooth functions of the surface orientations while the individual cubic harmonic function are smooth functions. Figure S6 shows how the value of an example GCN feature (GCN 4.8) changes non-smoothly while an example of a cubic harmonic function (L=13) changes smoothly as a function of the surface orientations. Since the individual GCN features are non-smooth functions, it is reasonable that the overall GCN model predictions are non-smooth. Similarly, since the individual cubic harmonic functions are smooth functions, it is reasonable that the overall cubic harmonics model predictions are smooth.

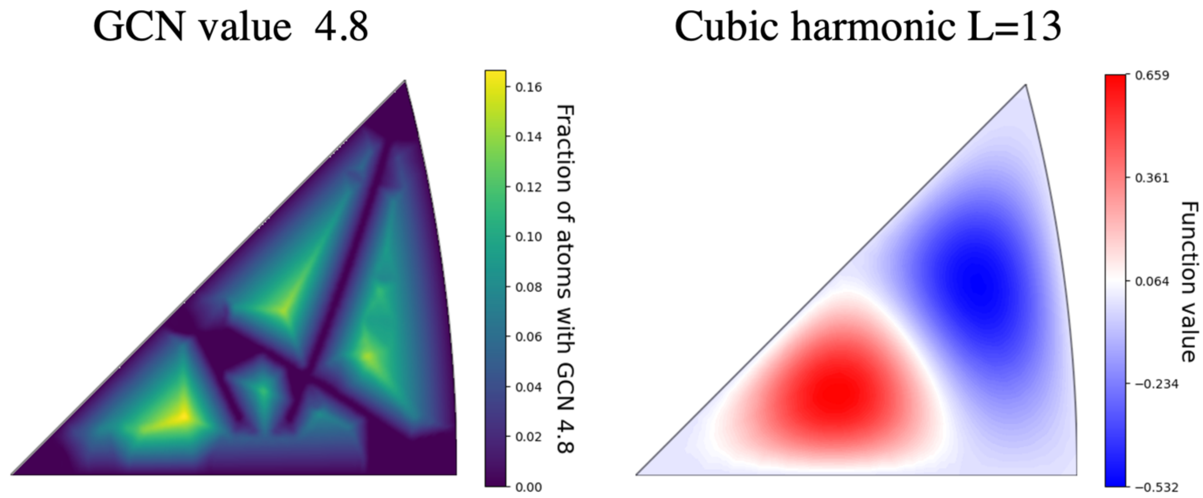

Figure S6: contour plot of the GCN value of 4.8 and the cubic harmonic function L=13 of a grid of surfaces that span the stereographic triangle. The GCN value 4.8 is a non-smooth function of the surface orientations with sharp change in its value while the cubic harmonic function is smooth.

7. Effect of changing the training data on the extrapolation of the models

We found that the magnitude of the model extrapolations to be sensitive to the different random splits of the data to train and test sets. To quantify the effect of changing the data splitting on the model extrapolations, we split the data random into a hundred different splits. For both the GCN and the cubic harmonics models, we trained hundred different versions of each model on a hundred different training splits. Figure S6 a, b show the distribution of the predicted maximum halftime difference using the GCN model and the harmonics models respectively. The mean value of the maximum predicted halftime difference by the GCN model is 1608 seconds. The mean value of the predicted maximum halftime difference by the cubic harmonics model is 1141 seconds. Figure S6 c shows the contour plot of the mean value of the predictions of the hundred GCN model versions for each surface orientation that span the stereographic triangle. All the hundred GCN models consistently predict the (11, 3, 1) surface orientation, highlighted by the black star, to have the highest enantiospecificity. Figure S7 d shows the contour plot of the mean value of the predictions of the hundred cubic harmonics model versions for each surface orientation that span the stereographic triangle. The hundred different models identified two different surface orientations, highlighted by the black stars, to have the highest enantiospecificity. Since these two surface orientations are located very close to the (17,5,2) surface orientation on the stereographic triangle, the location of the next experiment will not change as a result of this variance in the cubic

harmonics model predictions. We show here that even though the magnitude of the extrapolated predictions varies, the location of the optimal surface orientation in the stereographic triangle does not change significantly for both the GCN and the harmonics models.

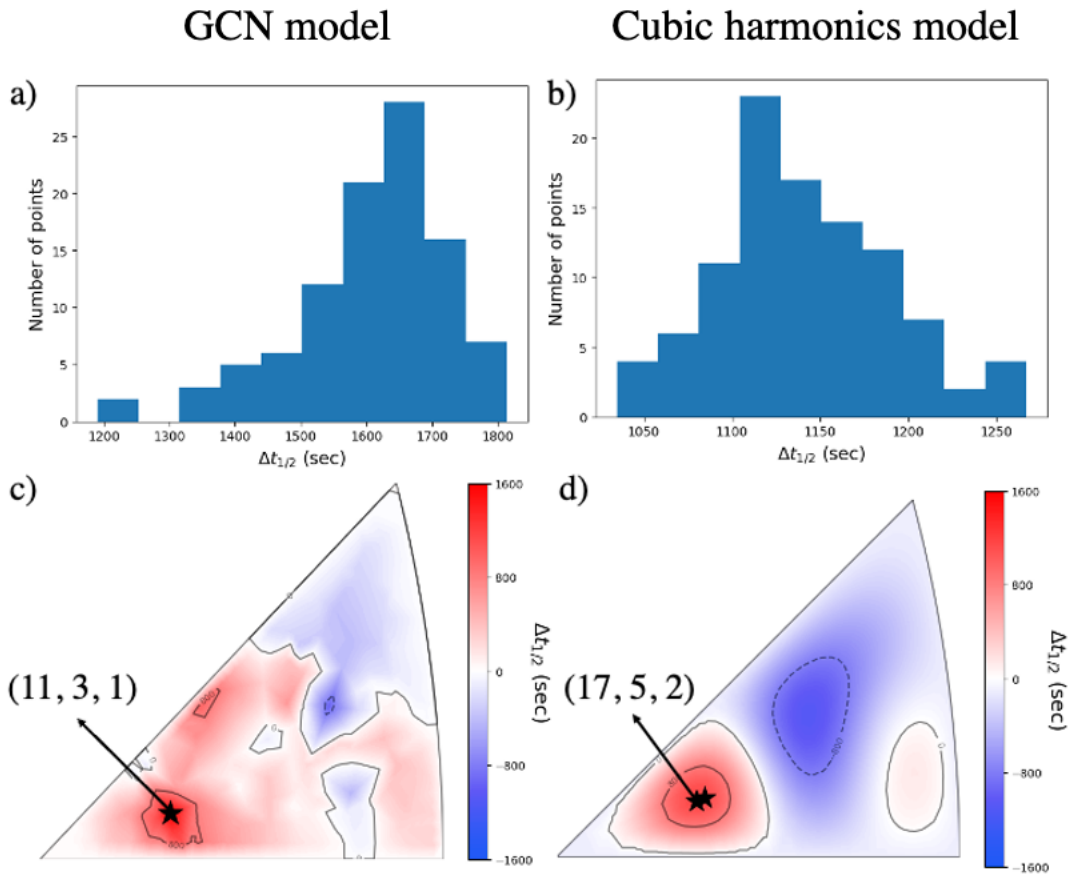

Figure S7: shows the effect of using a hundred different random splits of the data to train the models a, b) show the distribution of the predicted maximum halftime difference by GCN model and the harmonics models respectively. C) shows that all the hundred version of the GCN models consistently predict the (11, 3, 1) surface orientation to have the highest enantiospecificity. D) shows that the 100 versions of the harmonics model predicted surfaces that are located closely to the (17,5,2) surface orientation.

References

(1) Radetic, M. Surface Chemistry of Chiral Compounds on Chiral and Achiral Surfaces. thesis, Carnegie Mellon University, 2022. https://doi.org/10.1184/R1/19716295.v1.

(2) Virtanen, P.; Gommers, R.; Oliphant, T. E.; Haberland, M.; Reddy, T.; Cournapeau, D.; Burovski, E.; Peterson, P.; Weckesser, W.; Bright, J.; van der Walt, S. J.; Brett, M.; Wilson, J.; Millman, K. J.; Mayorov, N.; Nelson, A. R. J.; Jones, E.; Kern, R.; Larson, E.; Carey, C. J.; Polat, İ.; Feng, Y.; Moore, E. W.; VanderPlas, J.; Laxalde, D.; Perktold, J.; Cimrman, R.; Henriksen, I.; Quintero, E. A.; Harris, C. R.; Archibald, A. M.; Ribeiro, A. H.; Pedregosa, F.; van Mulbregt, P.; Contributors, S. 1 0. SciPy 1.0--Fundamental Algorithms for Scientific Computing in Python. *Nat. Methods* **2020**, *17* (3), 261–272. https://doi.org/10.1038/s41592-019-0686-2.

(3) Ahmadi, A.; Attard, G.; Feliu, J.; Rodes, A. Surface Reactivity at “Chiral” Platinum Surfaces. *Langmuir* **1999**, *15* (7), 2420–2424. https://doi.org/10.1021/la9810915.
